# Effect of auriculotherapy on labor pain severity and labor duration: a clinical trial

**DOI:** 10.61622/rbgo/2025rbgo33

**Published:** 2025-09-08

**Authors:** Somayehsadat Eslami, Omolbanin Heydari, Moghaddameh Mirzaee, Zahra Shad, Firoozeh Mirzaee

**Affiliations:** 1 Kerman University of Medical Sciences Kerman Iran Kerman University of Medical Sciences, Kerman, Iran.

**Keywords:** Auriculotherapy, Pregnancy, Labor pain, Pain management, Visual Analog Scale, Acupuncture points, Labor duration, Primiparous women

## Abstract

**Objective:**

Pain is an unavoidable reality of labor and the most noticeable determinant of the labor experience. The present study was conducted to assess the effect of auriculotherapy on labor pain and labor duration.

**Methods:**

This double blind, randomized, controlled clinical trial was conducted from 2021–2022 in Kerman, Iran. The sample consisted of 60 primiparous women assigned to an Intervention Group (*n* = 30) and a Control Group (*n* = 30). The study began after participants entered the active phase of labor at 37–40 weeks of pregnancy. In the Intervention Group, the researcher stimulated the desired points (uterus, pituitary gland, pelvis, cerebral, autonomous, sensory, endocrine gland, shen men, zero, external genitalia, and thalamus). Sham auriculotherapy (patches without seeds at the same acupoints) was done in the Control Group. Pain intensity in the two groups was recorded using the VAS before and after the intervention, when the cervix was dilated 4, 6, and 8 cm. Duration of labor was also recorded. Data were analyzed using SPSS 21 statistical software.

**Results:**

Pain intensity was significantly lower in the Intervention Group than in the sham Control Group (*P* < 0.0001) in different cervical dilatations after intervention. Compared to the sham Control Group, the intervention group was significantly different in the average duration of labor (*P* < 0.0001).

**Conclusions:**

Auriculotherapy was demonstrated to attenuate the severity of labor pains and expedite the labor duration. Hence, policymakers in this field are recommended to pay greater attention to this non-invasive method.

## Introduction

Labor pain is a multifactorial phenomenon influenced by physiological and psychological factors.^(1)^ Uterine contractions and cervical dilation are the primary physiological drivers, while maternal anxiety and fear contribute to the overall pain experience.^(2)^ Individual variations in maternal characteristics, such as age, parity, and physical condition, further modulate the intensity and duration of labor pain.^(3)^

The protracted nature of labor stages significantly influences perinatal outcomes.^(4)^ Prolonged labor is associated with increased pain, adverse obstetric outcomes, and elevated levels of fetal membrane inflammatory markers.^(5)^ Given the heightened risk of maternal morbidity and perinatal asphyxia, interventions that expedite labor progression are often considered.^(6)^

Despite the significance of labor pain management in obstetrics, there remains a paucity of standardized, adverse-effect-free analgesic strategies.^(1)^ Current approaches to labor pain relief predominantly fall into two categories: pharmacologic and non-pharmacologic interventions.^(7)^ While pharmacologic methods can offer adequate analgesia, they often carry potential risks to both the mother and fetus. Conversely, non-pharmacologic techniques, although generally well-tolerated, may provide variable levels of pain relief.^(8)^

Prolonged labor in primiparous women is characterized by a first-stage active phase lasting more than six hours.^(9)^ The labor process is divided into four stages, with the first stage comprising a latent and an active phase.^(10)^ Contractile dysfunction, particularly during the first and second stages, is a common cause of prolonged labor, leading to increased labor duration.^(11)^

Auriculotherapy, a form of acupressure, has shown potential in managing pain related to conditions such as low back pain, hip fractures, and gynecological disorders. While numerous studies have investigated its efficacy for these conditions, the evidence regarding its application for labor pain management remains equivocal, with reported outcomes varying across studies.^(12)^

While acupuncture's precise physiological and neurological underpinnings remain incompletely understood, its therapeutic efficacy in pain management is well-documented.^(13)^

The analgesic effects of auricular therapy are postulated to be facilitated by the body's endogenous opioid system and the stimulation of the hypothalamic-pituitary axis.^(14)^ An additional advantage of this method is its potential to stimulate physiological processes and modulate autonomic functions, including those related to the reproductive system.^(15)^

Auriculotherapy finds application in a variety of conditions, including menstrual disorders such as dysmenorrhea and metrorrhagia, lactation insufficiency, gastrointestinal disturbances, and acute diarrheal illnesses.^(16–18)^ Notably, given the similarities between uterine contractions during labor and menstrual cramps, auriculotherapy is often considered a therapeutic option for labor pain.^(19)^

Auriculotherapy has been associated with a significant reduction in the duration of the active phase, as evidenced by research studies.^(20)^ A hypothesized correlation exists between the intensity of labor contractions and the rate of cervical dilation. Stimulation of acupoints may modulate uterine contractions, potentially accelerating labor progression and reducing its overall duration.^(21)^

Setiawandari et al. ^(11)^ found that auricular acupressure on the uterine point can shorten the labor duration in primiparous women, so it can be used as alternative complementary care to prevent prolonged labor. Alimoradi et al.^(22)^ reported that ear and body acupressure effectively reduces labor pain and the duration of the active labor phase. According to their research, both ear and body acupressure efficiently alleviate labor pain; ear acupressure offers the added benefit of potentially shortening the active phase of labor.^(22)^

In a systematic review, Wang et al.^(23)^ found that auriculotherapy can be effective in relieving the pain of delivery. Zhu et al.^(15)^ demonstrated that auricular point sticking can effectively reduce labor pain and anxiety during the latent period of labor. The treatment offers quick relief and can be safely integrated into a comprehensive treatment plan.^(15)^

Auricular therapy offers potential benefits in managing obstetric dystocia by reducing the duration of the expulsive phase and alleviating labor pain, providing a non-invasive approach to pain management during childbirth.^(24)^ Despite the limited research investigating the role of auricular therapy in managing labor pain, this study aimed to assess its efficacy in alleviating discomfort during the active phase of childbirth with specific ear points.

Given the well-documented heightened vulnerability of primiparous women due to various stressors associated with first-time childbirth, this study aimed to evaluate the efficacy of this method with specific points in reducing labor pain and duration. One of this study's novelties was using a sham control group.

## Methods

**Figure 1:**
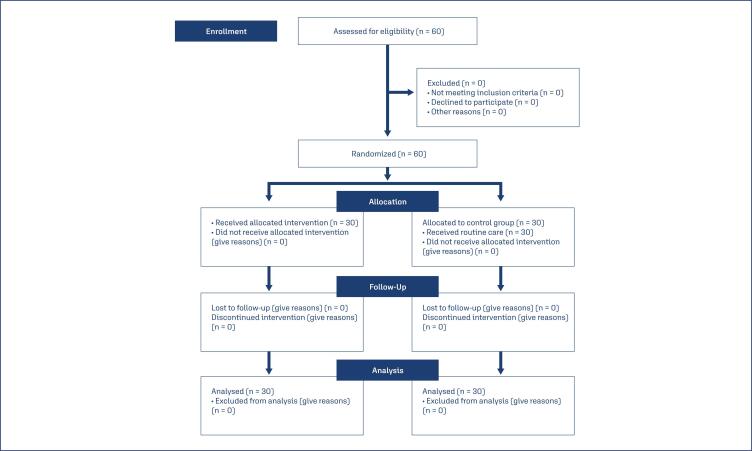
CONSORT flow diagram

This clinical trial was conducted from 2021–2022 on 60 primiparous women referred to Zarand Hospital, Kerman province, Iran. The participants were selected by referring to maternity hospitals and assessing the demographic and pregnancy information and eligibility of the pregnant women admitted. The data were collected by a midwife who was not on the research team. According to figure 1, sample size of 30 participants was calculated for each group (60 participants in the two groups) based on the study by Valiani et al.^(25)^ with a test power of 90% (β) and an error rate of 5% (α). (*d* = μ*1* - μ*2* = 9.25 - 8.35 ≈ 1, σ*1* = 1.33, σ*2* = 0.86, β = 0.9, α = 0.05)^(25)^

- Step 1 Randomization: In this double blind study, eligible patients were selected using the convenience sampling method. The patients were randomly allocated using random allocation software R version 4.3.2 to the auriculotherapy and sham control groups using simple random blocks.
- Step 2 Blinding: Because of the limitations of the intervention methods, we could not blind the researchers in the auriculotherapy group, but we could blind the participants in the auriculotherapy and sham control groups.

However, the statistician was blinded to reduce the risk of bias. In this regard, we defined the study groups as A and B for the statistician.

- Inclusion criteria: Low-risk pregnancy, gestational age of 37–40 weeks, cephalic presentation, single pregnancy, 4 cm dilatation, uterine contractions for 30 seconds and 2–3 contractions in 10 min, between 18–35 years of age, 18.5 < BMI < 24.9, basic literacy, and at least one healthy ear.
- Exclusion criteria: Taking painkillers 3 hours before and during the study, drug addiction, athletic activity, history of infertility, and the use of oxytocin to induce and accelerate labor.^(26)^

### Demographic questionnaire

The personal information questionnaire collected information on gestational age, education, income, mother's age, and husband's age.

### Visual analog scale (VAS)

#### Perceived pain in childbirth:

Visual analog scale (VAS): The visual analog scale is used to quantify the pain intensity, and it is often used to measure labor pain. Pain is measured with a 10 cm pain ruler and is one of the most common measures of pain. Participants were instructed to indicate their pain intensity on the visual analog scale (VAS).^(27)^This method is widely recognized for its reliability, sensitivity, and reproducibility in assessing pain severity. Moreover, its brevity and minimal language barriers facilitate cross-cultural comparisons. Among the various pain assessment instruments available, the VAS stands out for its consistent reliability and minimal measurement error.^(28)^

The validity was estimated at 0.97,^(29,30)^ with the same reliability as other research like, which used Cronbach's alpha to assess the reliability and reported a value of 0.86 with a 95% confidence interval of 0.81 to 0.90, demonstrating good reliability. The validity and reliability of VAS have been globally confirmed (*r* = 0.88).^(31,32)^ The participants were asked to describe their pain intensity before the intervention (4 cm) and at 6 and 8 cm dilations after the intervention, and the responses were recorded. Also, the duration of labor was measured from 4 cm dilation until delivery.^(33)^

**Figure 2:**
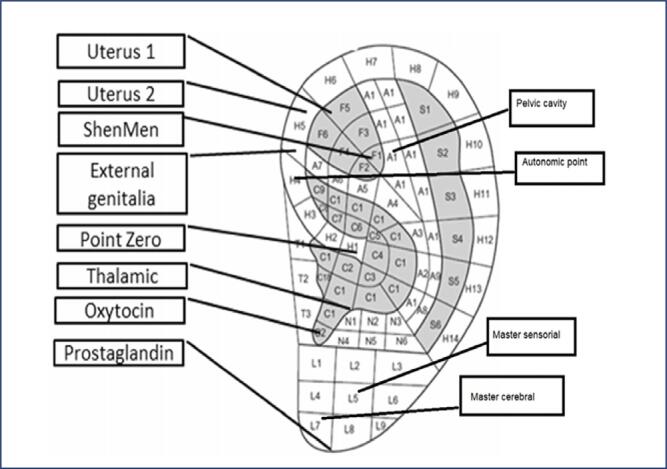
Points for auriculotherapy

The intervention group consisted of pregnant women at 37 to 40 weeks of pregnancy with 4 cm dilation of the cervix and 2–3 contractions over 10 minutes. Before the intervention, pain intensity at this dilation of the cervix was checked using the VAS. When the mother was not experiencing pain between contractions, after disinfecting one ear with 70% alcohol, the researcher stimulated the desired points (uterus, pituitary gland, pelvis, cerebral, autonomous, sensory, endocrine gland, shen men, zero, external genitalia, and thalamus) (Figure 2) on the external ear using Excel II (Thought Technology, Montreal, QC, Canada) and a manual probe made in China, and conducted seed treatment in these points. Seeds stimulate these points every thirty minutes for 5 to 10 seconds. The intensity of pain at 6 and 8 cm dilatations of the cervix was recorded by the researcher (SE), who is a midwife and an expert in auriculotherapy, between contractions. The duration of labor was also recorded.

In the sham Control Group, this group consisted of pregnant women at 37 to 40 weeks of pregnancy with a 4 cm dilation of the cervix and 2–3 contractions over 10 minutes. This group was treated like the Auriculotherapy Group, but the ear points were covered with patches without seeds. Before the intervention, pain intensity at 4 cm dilation of the cervix was checked with the VAS. Intensity of pain was recorded at 6 and 8 cm dilatations of the cervix between contractions. The duration of labor was also recorded.

Data were analyzed by PASW Statistics Ver. 23 (SPSS Inc., Chicago, IL, USA) using descriptive statistics such as frequency, percent, mean, and standard deviation, Fisher's exact test, chi-square test, and ANCOVA.

This randomized controlled clinical trial was approved by the Ethics Committee of Kerman University of Medical Sciences (ethics code IR.KMU.REC.1399.650) and registered under the clinical trial code IRCT20170624034722N1. Informed consent was obtained from participants, and they were assured they could withdraw from the study at any time.

## Results

No statistically significant difference was observed between the intervention and control groups regarding demographic variables, including gestational age, education, income, and husband's age (*P* > 0.05). According to table 1, most women in the study sample had achieved a high school diploma level of education and had a natural childbirth. The age group most frequently represented was 21–25 years old, and the participants predominantly belonged to the middle-income bracket (Table 1).

**Table 1:** Comparison of the demographic characteristics of the participants in groups

Variables		Intervention	Control	p-value
		n(%)	n(%)	
Type of	NVD	28(93.3)	22(73.3)	0.08*
delivery	C/S	2(6.7)	8(26.7)	
Gestational	37–38 W	5(16.7)	4(13.3)	0.88*
age	38–39 W	11(36.7)	13(43.3)	
	39–40 W	14(46.7)	13(43.3)	
Education	Under high-school diploma	7(23.3)	4(13.3)	0.55**
	High-school diploma	14(46.7)	14(46.7)	
	Higher than high-school	9(30)	12(40)	
	diploma			
Income	< 500 Dollars	12(40)	8(26.7)	0.61*
	500-1000 Dollers	16(53.3)	19(63.3)	
	> 1000 Dollars	2(6.7)	3(10)	
Mother's age	18–20	4(13.3)	4(13.3)	0.77*
	21–25	18(60)	15(50)	
	26–30	8(26.7)	11(36.7)	
Husband's age	21–25	10(33.3)	7(23.3)	0.42*
	26–30	18(60)	18(60)	
	31–35	2(6.7)	5(16.7)	

* Fishers exact test

** Chi-square test

Independent *t*-test was used to compare the intervention and control groups (before the intervention). ANCOVA (used to compare the two groups after intervention) revealed statistically significant disparities in terms of labor pain reduction after intervention (auriculotherapy) between the intervention and Control Group (*P* < 0.0001). According the results (Table 2), the participants in the Intervention Group demonstrated a significant decrease in labor pain after auriculotherapy. This decrease in pain was not seen in the Control Group, and the average severity of labor pain reported in the Control Group was higher than the Intervention Group in all dilatations (*P* < 0.0001). The greatest reduction in pain was seen in dilatations of 6 and 8. The most significant difference in the reduction of labor pain between the Control (7.93 ± 1.43) and Intervention group (5.60 ± 1.22) was seen in dilatation of 8 centimeters (Table 2).

**Table 2:** Comparison of labor pain severity in Auriculotherapy and sham Control Group

Variable		Auriculotherapy		Sham Control		p-value
		Mean	SD	Mean	SD	
Labor pain severity	Before intervention	3.73	1.14	5.20	0.99	< 0.0001*
	6 cm dilatation	4.93	1.25	6.80	0.99	< 0.0001**
	8 cm dilatation	5.60	1.22	7.93	1.43	< 0.0001**

* Independent t-test;

** ANCOVA test

Fisher's exact test revealed significant differences in labor duration between the intervention and control groups (Table 3). The participants in the intervention group experienced a shorter labor duration than the control group (*P* < 0.0001). The labor duration for most of the participants in the intervention group lasted less than 6 hours. In comparison, the duration of labor for most of the participants in the control group was more than 6 hours (Table 3).

**Table 3:** Comparison of labor duration in Auriculotherapy and sham Control Group

Variable		Auriculotherapy		Sham Control		p-value*
		No.	Percentage	No.	Percentage	
Labor duration	< 3 h	4	13.3	0	0.0	< 0.0001
	>6 h	21	70	8	26.7	
	> 6 h	5	16.7	22	73.3	

* Fisher's exact test

## Discussion

Given the paramount importance of pain management during childbirth, the absence of a universal, side-effect-free method remains a significant challenge in obstetrics. Prolonged labor, a known risk factor for adverse maternal and fetal outcomes, underscores the need for strategies to expedite delivery while minimizing discomfort. The results of our study showed that auriculotherapy can be effective in reducing the severity and duration of labor compared to the control group.

Our findings are consistent with the study of Alimoradi et al.,^(22)^ which indicates that auricular acupressure is highly efficacious in mitigating labor pain and decreasing the duration of the active phase of labor.

Consistent with the study of Xu et al.,^(24)^ the results of the present study also emphasize the effect of auriculotherapy on labor pain. Their meta-analysis indicates a potential for auricular acupressure to modulate the physiologic processes underlying labor, as evidenced by a reduction in the duration of the active phase and a trend towards shorter second and third stages. In this study, similar to our study, the greatest pain reduction was in high dilatations (6, 8, and 10 cm).^(24)^

In keeping with the study of Vakilian et al.,^(20)^ which was conducted to assess the effect of auriculotherapy during the active phase and the use of oxytocin during labor in nulliparous women, the results revealed that the length of the active phase indicated a significant reduction in the auriculotherapy group compared to the oxytocin group. Their study also confirms the effectiveness of auriculotherapy in reducing childbirth.^(20)^

Zhu et al.^(15)^ study on the effects of auricular point sticking on labor pain and anxiety proved auricular point sticking therapy can relieve anxiety and pain in women during the latent period of labor, which is consistent with the present study.

Consistent with the study of Setiawandari et al.,^(11)^ our research demonstrated a significant difference in labor duration after auriculotherapy. That study investigated the impact of the effect of auricular acupressure on uterine contractions, fetal heart rate, and length of labor in the active phase of primiparous women, indicating the significant effect of auriculotherapy on shortening the first stage of the active phase.^(11)^

Also, our findings are consistent with the study of Ho et al.^(34)^ on the effects of multi-mechanism complementary therapy on pain and anxiety during labor latency in primiparous women. In this study, which was a multi-intervention research, auriculotherapy was one of the non-pharmacological methods of reducing labor pain and anxiety, and its effect was investigated. In their study, auriculotherapy significantly affected the relief of labor pain, which is in line with our findings.^(34)^

Our study is comparable to the literature, including the study of Oviedo et al.^(35)^ on pain management during first-trimester abortion, Prakash et al.^(36)^ on pain relief in post-cesarean women by percutaneous stimulation of auricular pressure points, and Mousavi et al.^(37)^ on the comparison of two methods of complementary medicine on postoperative pain.

Non-pharmacological pain relief methods for childbirth are accessible, inexpensive, and easy to implement. The lack of these methods is a telltale sign of inadequate care and is associated with fewer women giving birth in healthcare facilities, ultimately impacting maternal health outcomes.^(38)^

A variety of pharmacological and non-pharmacological interventions are currently employed to alleviate the discomfort associated with labor. These methods exhibit varying degrees of efficacy in reducing labor pain. Auriculotherapy distinguishes itself from other pain management strategies due to its simplicity. Moreover, this approach has demonstrated comparable effectiveness to other modalities.^(19)^

One of the strengths of this study was the use of a sham control group, which allowed for control of confounding factors. Patches without seeds at the same acupoints can be used as a sham control method for the ear acupressure procedure. This study had several limitations. Differences in the mental-psychological conditions of the participants, their perception of childbirth, different pain thresholds (people subjected to the same pain may have different perceptions of pain), as well as varying responses of the cervix to uterine contractions were among the limitations of this study. This pain difference could be attributed to individual differences, economic status, and cultural factors. Therefore, attempts were made to control this limitation through randomization. The small sample size and limited statistical population were other limitations of this research.

## Conclusion

Auriculotherapy has been demonstrated to attenuate the severity of labor pains and expedite the labor duration. Hence, policymakers in this field are recommended to pay greater attention to this non-invasive method.
